# Successful simultaneous laparoscopic repair of concurrent diaphragmatic eventration, hiatal hernia, and recurrent inguinal hernia in a single procedure: a case report

**DOI:** 10.3389/fsurg.2026.1748082

**Published:** 2026-02-09

**Authors:** Chenjie Gao, Yansong Pu, Bin Song

**Affiliations:** The Second Department of General Surgery, Shaanxi Provincial People’s Hospital, Xi'an, Shaanxi, China

**Keywords:** diaphragmatic eventration, hiatal hernia, inguinal hernia, multiple hernias, one-stage repair

## Abstract

This article presents the case of a 59-year-old female patient with left-sided diaphragmatic eventration, hiatal hernia, and recurrent left inguinal hernia. A carefully planned single laparoscopic procedure was developed to carry out partial diaphragmatic resection with mesh reinforcement, hiatal hernia repair with Nissen fundoplication, and tension-free inguinal hernia repair, all based on detailed preoperative imaging evaluation. The innovative aspects of this procedure include: (1) a simultaneous transabdominal approach for the management of diaphragmatic eventration and multiple hernias; (2) the use of a linear stapler for partial diaphragmatic resection to reduce suture tension; and (3) reinforcement of the weakened diaphragmatic area with a double-layer mesh. The patient recovered well postoperatively without complications. The goal of this example is to give a new view of the minimally invasive care of complicated multiple hernias.

## 1 Introduction

Diaphragmatic eventration is primarily a congenital condition characterized by underdevelopment of the diaphragmatic muscle, leading to abnormal elevation of the diaphragm (1). It is frequently credited with traumatic reasons in adults (2). Common symptoms include dyspnea, cough, wheezing, and gastrointestinal discomfort such as epigastric fullness, belching, nausea, and vomiting. A hiatal hernia occurs when abdominal contents protrude through the esophageal hiatus into the thoracic cavity⁠ (3), resulting in gastroesophageal reflux-related symptoms including acid reflux, heartburn, and chest pain. The coexistence of diaphragmatic eventration and hiatal hernia is exceedingly rare, with few cases documented in the literature (4, 5). This patient had a previous history of trauma, followed by symptoms suggestive of diaphragmatic eventration such as chest tightness and wheezing, which were initially overlooked. Recently, symptoms of acid reflux and heartburn developed, leading to hospital examination. CT scans were performed on upper gastrointestinal radiography and gastroscopy verified that there was diaphragmatic eventration in addition to a hiatal hernia. This rare presentation of acquired diaphragmatic eventration associated with hiatal hernia complicates both diagnosis and treatment. Previous animal studies have suggested that repairing diaphragmatic eventration may weaken the anti-reflux barrier and exacerbate gastroesophageal reflux symptoms⁠ (6). Diaphragmatic eventration is typically repaired via thoracic surgery (7), while hiatal hernia repair with anti-reflux surgery is commonly performed laparoscopically⁠ (8). Addressing the diaphragmatic eventration first could worsen gastroesophageal reflux and necessitate multiple operations, increasing the patient's hospitalization and treatment duration. In addition, in this instance, the possibility of a recurrent left inguinal hernia led to the choice to perform a simultaneous laparoscopic repair of the left diaphragmatic eventration, the hiatal hernia, and the recurrent left inguinal hernia; this procedure was performed in a single operation.

## 2 Medical history and treatment course

### 2.1 History and examination

**Figure 1:**
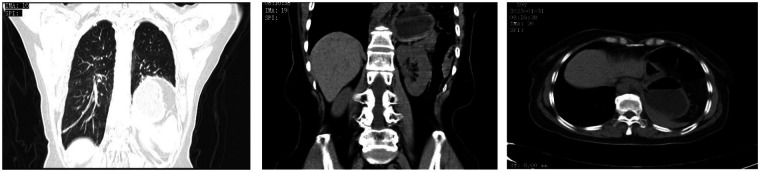
The CT scan showed left diaphragmatic eventration and a hiatal hernia.

**Figure 2:**
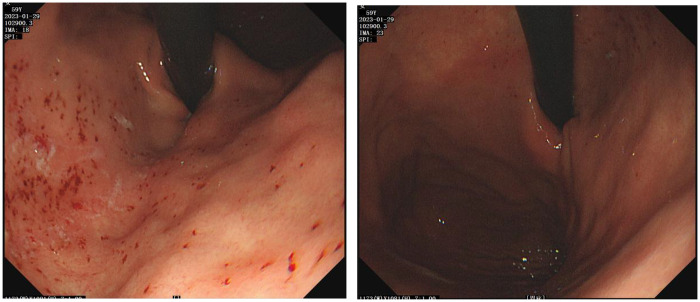
Endoscopy indicated chronic gastritis and hiatal hernia.

**Figure 3:**
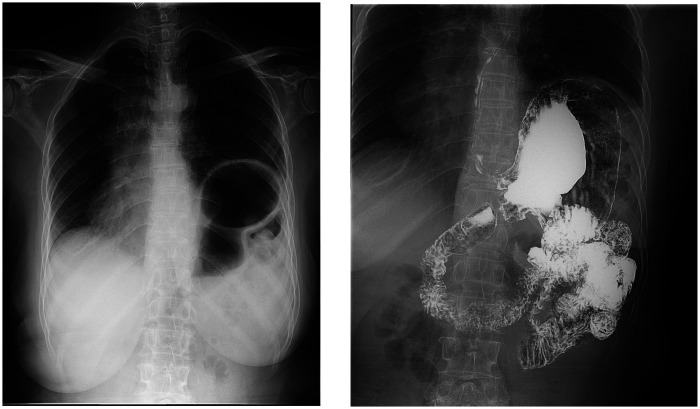
Upper gastrointestinal series: elevated left hemidiaphragm.

A 59-year-old female presented with a six-month history of postprandial abdominal pain and bloating, accompanied by worsening acid reflux and nausea. Her surgical history included two previous left inguinal hernia repairs (32 years and 18 years ago, both open surgeries) and left chest trauma two years prior. CT images showed bilateral pulmonary exudative changes, left diaphragmatic eventration, and hiatal hernia (Figure 1). Gastroscopy revealed chronic atrophic gastritis (Figure 2), and 24-h esophageal pH monitoring corroborated the presence of pathological acid reflux. The upper gastrointestinal series showed an elevation of the left hemidiaphragm (Figure 3).

### 2.2 Surgical indications

**Figure 4:**
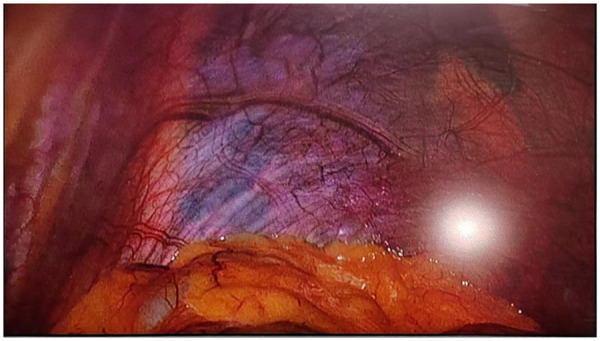
Laparoscopic exploration revealed elevation of the left diaphragm.

**Figure 5:**
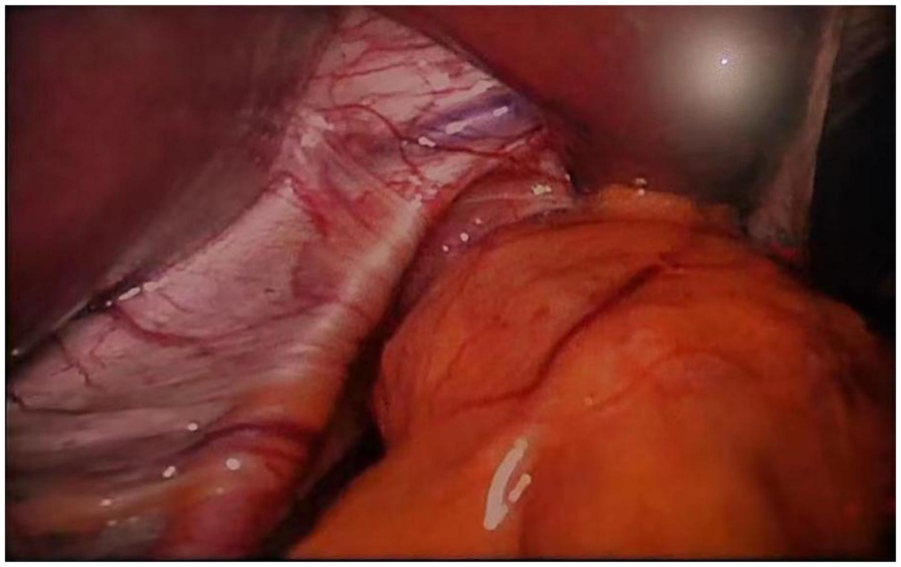
Laparoscopic observation of hiatal hernia.

**Figure 6:**
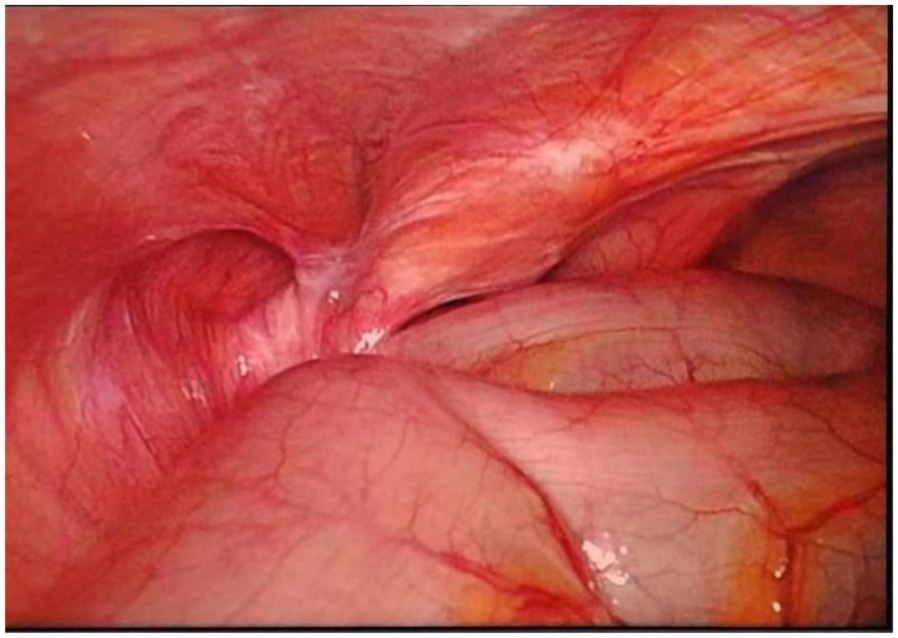
Laparoscopy revealed a left inguinal hernia.

(1). Left diaphragmatic eventration (Figure 4); (2). Hiatal hernia (Figure 5) with gastroesophageal reflux disease; (3). Recurrent left inguinal hernia requiring repair (Figure 6).

### 2.3 Surgical plan

The following criteria served as the basis for the design of the surgical strategy:

1. **Approach selection**: Perform all procedures—diaphragmatic, hiatal, and inguinal—through a single laparoscopic approach to minimize surgical trauma.
2. **Management of diaphragmatic eventration**: A modified Kimura procedure using a linear stapler to resect the lax portion of the diaphragm, combined with reinforcement using an anti-adhesion coated polypropylene mesh (15 cm × 10 cm)
3. **Anti-reflux** procedure: Narrowing of the diaphragmatic crura, reinforcement with biologic mesh, and Nissen fundoplication (360° wrap).
4. **Inguinal hernia repair**: A TAPP approach was used to place a mesh inside the preperitoneal area.

### 2.4 Surgical procedure

**Figure 7:**
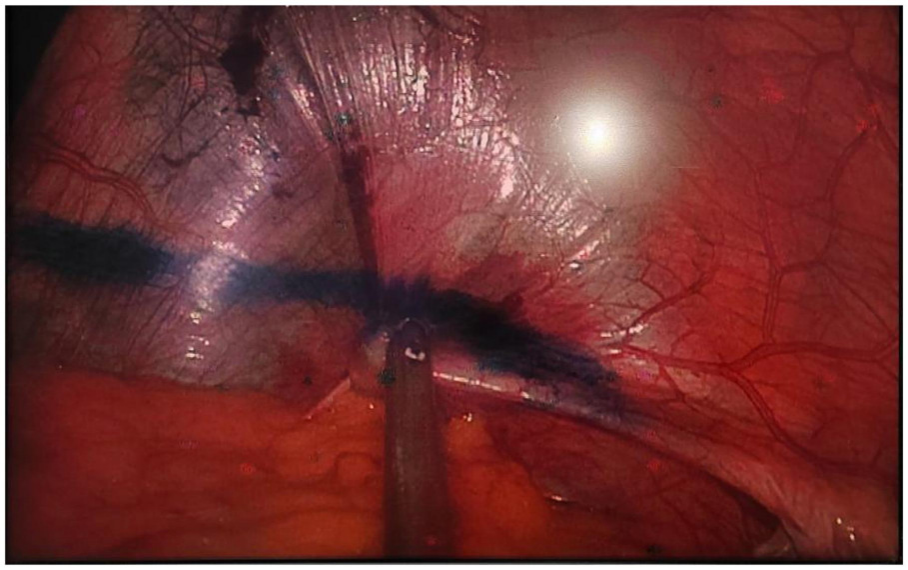
Mark the direction from the cardiophrenic angle to the costophrenic angle with methylene blue.

**Figure 8:**
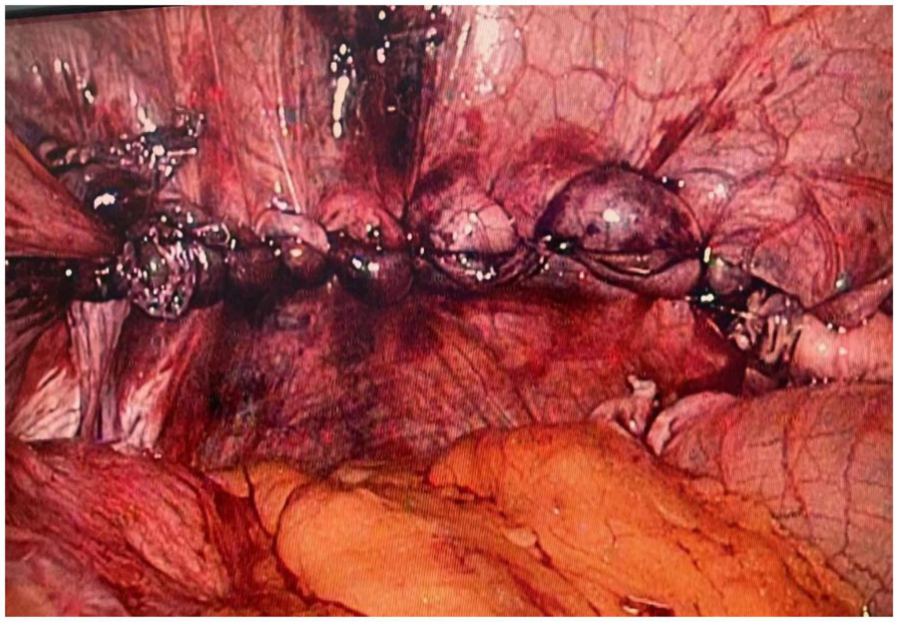
The redundant diaphragm is resected using a linear cutting stapler, followed by continuous imbrication with barbed sutures.

**Figure 9:**
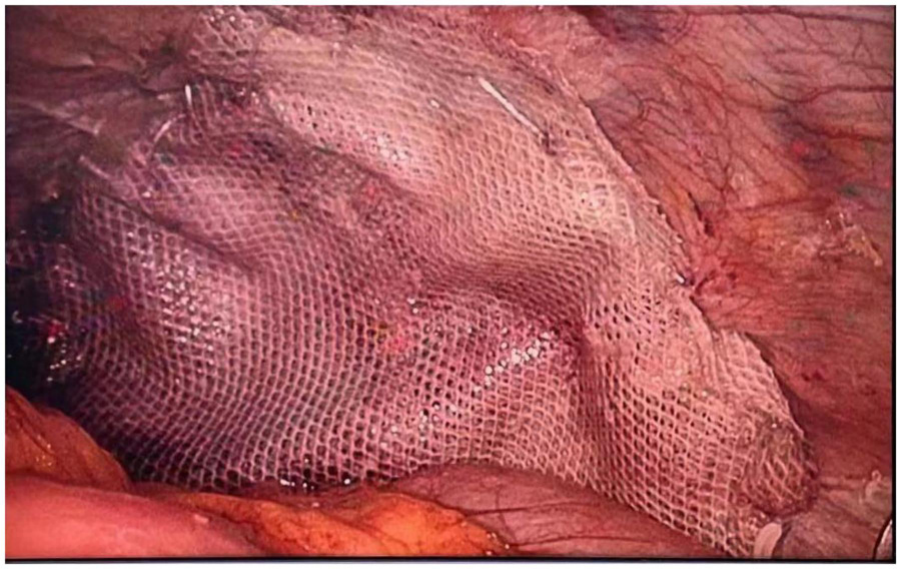
The double-sided mesh is secured with adhesive after covering the diaphragm, and the periphery of the mesh is continuously sutured and fixed with barbed sutures.

**Figure 10:**
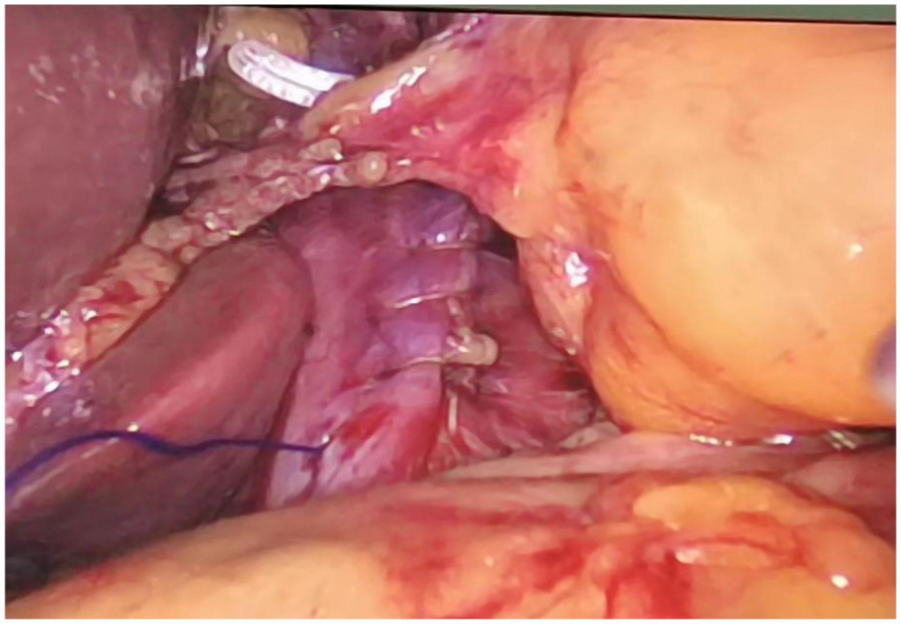
Narrowing of the diaphragmatic crura.

**Figure 11:**
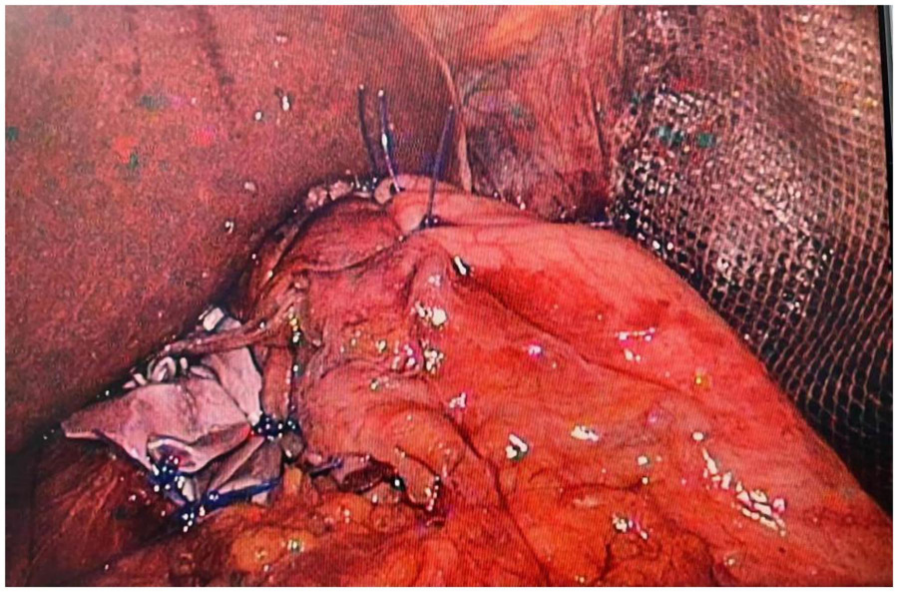
Reinforcement of the diaphragmatic crura with biological mesh and nissen fundoplication.

(1) **Positioning and Trocar Placement**: The patient was placed in a supine position with the left side elevated by 30° and in a reverse Trendelenburg position. A 10 mm observation port is placed above the umbilicus, and 12 mm, 12 mm, and 5 mm operation ports are placed below the left costal margin, outside the right rectus abdominis, and below the right costal margin, respectively, with a pneumoperitoneum pressure of 13 mmHg.
(2) **Repair of Diaphragmatic Eventration**: ①. The direction from the cardiophrenic angle to the costophrenic angle was marked with methylene blue (Figure 7); ②. Redundant diaphragmatic tissue was resected using a linear stapler; ③. Continuous imbricating sutures were applied using barbed suture (Figure 8); ④. An anti-adhesion coated polypropylene mesh was applied to cover the weakened area and fixed with adhesive and sutures (Figure 9).
(3) **Management of Hiatal Hernia**: ①. The diaphragmatic crura were freed and the hiatus narrowed to approximately 1 cm with interrupted 2-0 Prolene sutures (Figure 10); ②. A U-shaped biologic mesh was placed over the hiatus and fixed with a spiral tacker; ③. Perform Nissen fundoplication (360° wrap, 2 cm esophageal segment) (Figure 11).
(4) **Inguinal Hernia Repair**: ①. Open the preperitoneal space and free the Retzius and Bogros spaces; ②. To coat the myopectineal orifice, a mesh was inserted, and it was fixed with biologic adhesive.

### 2.5 Postoperative care and follow-up

**Figure 12:**
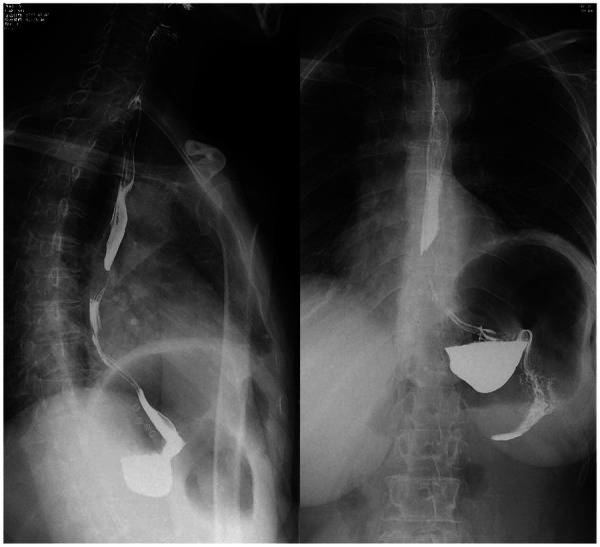
Upper gastrointestinal series: No significant gastroesophageal reflux was observed, and the diaphragmatic bulging was markedly improved compared to previous findings.

On postoperative day 1, small-volume water was given repeatedly; after confirming no obvious discomfort and normal drain output, the abdominal drain was removed. A liquid diet was started on day 3. Because multiple meshes were implanted and the operation lasted >2 h, cefoxitin sodium was administered pre-, intra- and postoperatively to prevent mesh infection. A six-month postoperative follow-up and re-examination via upper gastrointestinal angiography were conducted on the patient. The patient exhibited no symptoms of chest tightness or shortness of breath, and no discomfort such as nausea, vomiting, acid reflux, heartburn, abdominal pain, or abdominal distension after eating. Upper gastrointestinal angiography revealed no evidence of reflux, and the diaphragmatic eventration exhibited a substantial improvement in comparison with its preoperative state (Figure 12). A physical examination was conducted, revealing no discernible bulge protrusion in the left inguinal region, indicating no recurrence of the left inguinal hernia.

## 3 Discussion

### 3.1 Innovations compared to traditional suturing techniques

The overlapping suture technique of the diaphragm, known as the Kimura technique (9), creates an overlapping structure through full-thickness folding sutures without incising the diaphragm. While this technique can enhance tension, it presents two primary concerns: ① it does not remove redundant diaphragmatic tissue, and reliance solely on suture traction can easily result in uneven local tension; ② multi-layer folding may compress branches of the phrenic nerve, thereby affecting postoperative diaphragmatic motor function. In this case, a linear cutting stapler is employed to directionally resect the bulging diaphragm, directly eliminating tissue redundancy, avoiding mechanical imbalances caused by folding traction, and simultaneously reducing the risk of nerve damage.

The diaphragmatic overlapping suture technique, also known as the Maxson technique (10), involves incising the diaphragm into upper and lower layers, followed by suturing them in an overlapping manner. While this method can result in a thicker diaphragmatic layer, it necessitates a full-thickness incision of the diaphragm, which may compromise the continuity of thoracoabdominal pressure transmission. In this case, after excising redundant diaphragmatic tissue using a linear cutting stapler, the weakened area was reinforced with an anti-adhesion coated polypropylene mesh. This method not only preserves the anatomical integrity of the diaphragm but also achieves mechanical compensation through the elastic modulus matching of the mesh, making it more aligned with the principles of physiological reconstruction than basic suturing techniques.

Diaphragmatic plication, specifically the Eitan technique (11), involves the creation of folded layers through continuous suturing. However, this method may result in the loosening of the folded structure due to the attenuation of suture tension. To address this issue, a mesh reinforcement was employed, with the edges of the mesh being continuously sutured and secured to the diaphragm. This approach enhanced the strength of the diaphragm while preserving its mobility.

### 3.2 Complementary approach to partial resection involving end-to-end anastomosis

Traditional partial resection with end-to-end anastomosis is appropriate for localized eventration of the diaphragm; however, it may be inadequate in cases of extensive diaphragmatic weakness. The innovation of this case includes: ①. The use of a linear cutting stapler to perform extensive diaphragmatic resection, effectively removing the bulging area; ②. the application of a double-layer mesh (polypropylene with an anti-adhesion coating) to cover the wound, which not only compensates for the tissue defect following resection but also mitigates the continuous traction on the diaphragm caused by abdominal pressure through the tensile strength of the mesh. Compared to simple resection and suturing, the introduction of the mesh enhances the mechanical reconstruction of the diaphragm from “passive traction” to “active support”.

### 3.3 Individualized mesh selection rationale for multiple hernia repairs

The patient had multiple hernias, and a bespoke mesh was selected for each repair primarily based on the patient's individual conditions (including age and hernia type) and the team's previous clinical experience. Details, categorized by hernia type, are elaborated below. First, for the repair of traumatic diaphragmatic eventration, a polypropylene mesh with an anti-adhesion coating was chosen over a biodegradable mesh. This mesh was selected for its superior tensile strength and capacity to withstand pressure during thoracoabdominal activities (e.g., coughing or strenuous exercise), thereby avoiding suture margin tearing. Its non-absorbable properties ensure long-term structural integrity of the diaphragmatic defect and reduce the recurrence risk associated with age-related muscle atrophy and diaphragmatic relaxation. Additionally, the anti-adhesion coating minimizes adhesion to adjacent abdominal organs, further improving surgical safety. Second, biological mesh is preferred for hiatal hernia repair. This preference is based on our previous experience with double-layer meshes for hiatal hernia repair, where a small number of esophageal fistula cases were observed during follow-up. Presumably, this complication resulted from repeated friction between the rigid mesh surface and the peristaltic esophagus. Furthermore, follow-up data from our previous patients demonstrated no significant difference between biological and double-layer meshes in controlling gastroesophageal reflux symptoms or reducing recurrence rates. Third, regarding inguinal hernia repair, the patient had experienced recurrence following two prior open anterior approach surgeries, which conferred an extremely high risk of re-recurrence. We therefore opted for a non-absorbable mesh with high tensile strength to further minimize the recurrence risk. The surgical technique was modified to transabdominal preperitoneal (TAPP) repair to avoid adhesions and anatomical disruption caused by previous surgeries, thereby minimizing iatrogenic injury. This approach also eliminates the need for additional incisions, shortens operative time and reduces the likelihood of mesh infection. Moreover, the extensive mesh coverage achieved with TAPP repair reduces the risk of subsequent hernias in other groin regions, such as femoral and suprapubic hernias.

### 3.4 Breakthrough in instrument integration with minimally invasive techniques

Although thoracoscopic and laparoscopic diaphragmatic plication have become mainstream techniques, traditional minimally invasive procedures predominantly rely on manual suturing. The innovation presented in this study is twofold: ① the directional application of a linear cutting stapler allows for simultaneous diaphragmatic resection and hemostasis (with blood loss limited to less than 50 ml), significantly enhancing efficiency compared to traditional electric hook separation; ② the implementation of a “360° anchoring method” for mesh fixation involves continuous suturing at the edges of the diaphragm, costophrenic angle, and other margins. This creates a 360° mechanical anchoring zone that aligns more closely with biomechanical transmission principles and does not impede diaphragmatic movement.

## 4 Conclusion

This case advances diaphragmatic repair from “morphological restoration” to “functional remodeling” by integrating the device innovation and mechanical reconstruction. Understanding these abnormal anatomical and pathophysiological changes aids in formulating appropriate surgical approaches and management strategies.

## Data Availability

The original contributions presented in the study are included in the article/Supplementary Material, further inquiries can be directed to the corresponding author.
